# Weight Adaptive Path Tracking Control for Autonomous Vehicles Based on PSO-BP Neural Network

**DOI:** 10.3390/s23010412

**Published:** 2022-12-30

**Authors:** Xianzhi Tang, Longfei Shi, Bo Wang, Anqi Cheng

**Affiliations:** Hebei Key Laboratory of Special Delivery Equipment, School of Vehicles and Energy, Yanshan University, Qinhuangdao 066004, China

**Keywords:** autonomous vehicles, path tracking control, particle swarm optimization, model predictive control

## Abstract

In order to improve the tracking adaptability of autonomous vehicles under different vehicle speeds and road curvature, this paper develops a weight adaptive model prediction control system (AMPC) based on PSO-BP neural network, which consists of a dynamics-based model prediction controller (MPC) and an optimal weight adaptive regulator. Based on the application of MPC to achieve high-precision tracking control, the optimal weight under different operating conditions obtained by automated simulation is used to train the PSO-BP neural network offline to achieve online adjustment of MPC weight. The validation results of the Prescan-Carsim-Simulink joint simulation platform show that the adaptive control system has better tracking adaptation capability compared with the original classical MPC control. The control strategy was also verified on an autonomous vehicle test platform, and the test results showed that the adaptive control strategy improved tracking accuracy while meeting the vehicle’s requirements for real-time control and lateral stability.

## 1. Introduction

In recent years, with the increasing concern for autonomous driving and traffic safety, path tracking control, as a key technology in autonomous driving, has gradually become a hotspot for scholars at home and abroad to research [1]. However, when the vehicle is driving at medium to high speed on a large curvature curve, the vehicle will deviate from the intended driving path due to inertia, making the tracking accuracy unable to meet the requirements. This type of situation greatly limits the technological progress of autonomous vehicles [2,3].

Autonomous vehicle path tracking control focuses on controlling autonomous vehicles to follow the desired trajectory [4,5] or road centerline with high accuracy while satisfying stability [6]; a series of studies have been conducted by scholars on path tracking control techniques for autonomous vehicles, which can be broadly classified into three categories: (1) Geometry-based control methods, including pure control algorithm [7], Stanly tracking algorithm [8], etc. Some scholars have made improvements based on such algorithms to improve the tracking performance by adaptively adjusting the look-ahead distance [9,10,11], however, this type of method does not follow the parameter regulation method of the control theory system, so it is difficult to achieve the trade-off between stability and tracking accuracy [12]. (2) Feedback control without prediction, including sliding mode control [13,14], backstepping control [15,16], etc. This approach is based on a vehicle dynamics model to design a feedback control law to bring the system to a steady state; Hu, C. et al. proposed the integral sliding mode control to convert the path tracking control problem into a yaw stability control problem [17], and Wang, Y. Y. et al. proposed an adaptive backstepping sliding mode controller combining sliding mode control and backstepping control to obtain the desired velocity using the backstepping technique and an adaptive sliding mode approach to handle the unknown model uncertainty [18]. Although the effectiveness of the above control algorithms has been demonstrated by simulation validation or vehicle tests, the tracking accuracy of such methods in high-frequency and high-perturbation environments is not satisfactory due to the lack of prediction of future states and the identification of external disturbances. (3) Feedback control with prediction, including linear quadratic regulator (LQR) control [19], model predictive control (MPC) [20,21,22], etc. Among them, the LQR is favored by industry for its simple design and superior overall performance. However, when tracking curves with different curvatures, feedforward control needs to be introduced to achieve theoretical error-free tracking, yet adding feedforward control makes the controller sensitive to discontinuities in the reference trajectory, and additional adjustments are needed to weaken the sensitivity [12]. Compared with LQR, MPC performs rolling optimization in a limited time domain, which is well-suited to optimization problems with multiple constraints, at the same time MPC takes into account the future time domain driving road conditions, which also makes the algorithm more effective when tracking curves. However, MPC operation needs to be paired with high arithmetic hardware, so linear time-varying (LTV) MPC is mostly used in real vehicle control strategies [23,24].

The above studies provide rich path tracking control strategies, but with the change in environment and the vehicle’s own state, it makes the control algorithm with a good performance face the problem of tracking accuracy degradation in some severe situations, mainly because the control method with fixed parameters cannot adapt to the time-varying nature of the global environment; for such problems, using the prediction horizon as a real-time optimization variable to improve the algorithm’s adaptability is one of the mainstream directions of current research [25,26,27], but this approach makes it difficult to guarantee the algorithm’s real-time performance in some cases where vehicle stability needs to be prioritized, because the adaptive growth of the prediction horizon will make the matrix dimension increase and the amount of operations surge. Shan, Y. X et al. proposed an adaptive path tracking scheme by integrating reinforcement learning and combined model (PP-PID) to adjust the weights of PP-PID adaptively to balance their effects laterally according to the range of tracking error, lateral acceleration and steering angle variations. Moreover, a multi-layer speed adaptation method with fuzzy control is proposed for speed control to optimize the lateral tracking performance. The performance of the proposed tracking scheme was experimentally verified [28], but this scheme is only suitable for point-to-point autonomous driving tasks because the lateral control effect depends on the algorithm’s adaptive desired vehicle speed, which is not directly mapped as an element to the PP-PID parameters and cannot be regulated by subjective speed regulation through the driver. Lin, F. et al. performed online optimization of the prediction horizon, control horizon and sampling time of MPC algorithm based on vehicle speed [29]. However, Lin, F. et al. only divided the parameters to be optimized into three groups based on low, medium and high vehicle speed correspondence after qualitative analysis of the parameters, which could not meet the adaptive requirements under complex conditions. Zhang, K. et al. considered this from another perspective and proposed an ensemble-based parameter estimator through a machine learning-based approach and used the estimated system parameters in MPC to improve the control accuracy [30]. However, Zhang, K. et al. demonstrated the closed-loop stability of the algorithm only by numerical derivation and have not yet verified the effectiveness by real vehicles.

In response to the above deficiencies, in this paper, a weighted adaptive path tracking controller based on PSO-BP (Particle Swarm Optimization Back Propagation) neural network algorithm is proposed by considering the road factors and vehicle speed in driving and using automated simulation and tuning techniques, with the following main contributions: (1) Automated simulation tests are used to obtain optimal parameter combinations under different conditions, produce a dataset, propose a PSO-BP neural network and train the weight adaptive model offline. The purpose of this is to prevent the model training from falling into local optimum and to fit the nonlinear relationships that are difficult to map. (2) The effect of MPC algorithm weights on the tracking performance when the vehicle is driving normally on a structured road is analyzed. The weight is adaptively adjusted by the model based on the current vehicle speed and road curvature to improve the adaptability of the vehicle in different conditions. (3) A vehicle development-friendly Hildreth real-time solver was used to conduct a structured open-road vehicle test, which experimentally verified the performance of the algorithm.

The rest of this paper is organized as follows: Section 2 derives the vehicle dynamics model applied in this paper. Section 3 performs the design of the model prediction controller including the real-time solver. Section 4 constructs a weight adaptive control strategy based on PSO-BP neural network. Section 5 conducts simulations and analyzes results based on the joint Prescan-Carsim-Simulink simulation platform. Section 6 conducts and analyzes the vehicle tests. Finally, the conclusions are given in Section 7.

## 2. Vehicle Dynamics Model

The vehicle is a highly complex inertial system that is difficult to model for accurate description. In order to build a simplified model, the following reasonable assumptions are made before modeling for the problem under study.

- The vehicle is always driven on a flat surface;
- Ignore the suspension action and the vehicle’s vertical motion;
- Ignore the effect of the vehicle’s own steering structure;
- The vehicle’s left- and right-side tires are consistent;
- Ignore the air resistance of the vehicle body.

**Figure 1:**
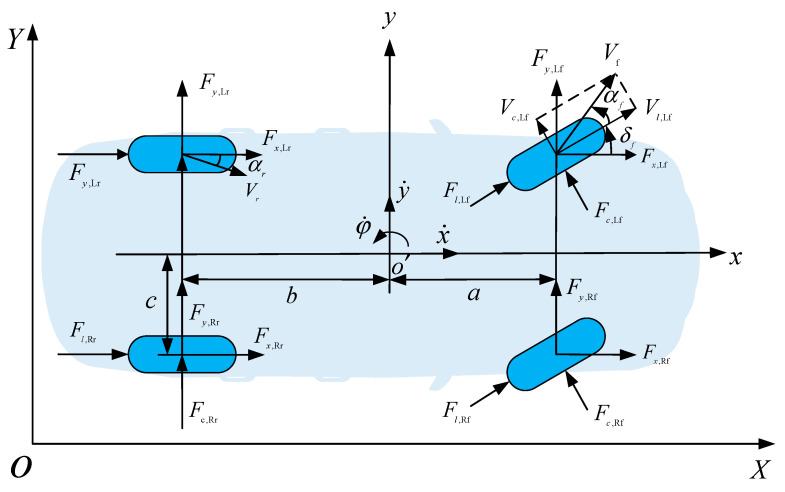
Vehicle dynamics model.

In this paper, we select three degrees of freedom in x,y directions and rotation around z axes to establish a simplified three-degree-of-freedom dynamics model of the vehicle with four wheels. Figure 1 shows a bicycle model of a vehicle. The XOY coordinate system denotes the geodesic coordinate system and the xoy coordinate system denotes the vehicle coordinate system, both of which satisfy the right-handed spiral rule. The symbolic definitions of the physical quantities in dynamics are shown in Table 1.

Applying Newton’s Second Law to longitudinal, lateral, and yaw degrees of freedom, vehicle dynamics model can be constructed:

(1) $$\begin{array}{l} m\ddot{x}=m\dot{y}\dot{\varphi }+F_{x,\mathrm{Lf}}+F_{x,\mathrm{Rf}}+F_{x,\mathrm{Lr}}+F_{x,\mathrm{Rr}}\\ m\ddot{y}=m\dot{x}\dot{\varphi }+F_{y,\mathrm{Lf}}+F_{y,\mathrm{Rf}}+F_{y,\mathrm{Lr}}+F_{y,\mathrm{Rr}}\\ I_{z}\ddot{\varphi }=a\left(F_{y,\mathrm{Lf}}+F_{y,\mathrm{Rf}}\right)-b\left(F_{y,Lr}+F_{y,Rr}\right)+\\ c\left(F_{x,Lr}+F_{x,Rr}-F_{x,\mathrm{Rf}}-F_{x,\mathrm{Lf}}\right) \end{array}\}$$

where $\ddot{x}$, $\ddot{y}$ are the longitudinal acceleration and lateral acceleration of the vehicle in the vehicle coordinate system, respectively. The transformation relationship between coordinate system xoy and coordinate system XOY is:

(2) $$\begin{array}{l} \dot{X}=\dot{x}\cos\varphi -\dot{y}\sin\varphi \\ \dot{Y}=\dot{x}\sin\varphi +\dot{y}\cos\varphi \end{array}$$

$\dot{X}$, $\dot{Y}$ are the velocity components along the horizontal and vertical coordinates in the geodetic coordinate system, respectively.

Under regular driving conditions and when the longitudinal sliding rate is small, the longitudinal force of the tire is proportional to the sliding rate, and the longitudinal force of the tire in this case can be described as:

(3) $$\begin{array}{l} F_{x,\mathrm{Lf}}=C_{lf}s_{f}\\ F_{x,\mathrm{Lr}}=C_{l_{r}}s_{r} \end{array}\}$$

where $C_{lf}$, $C_{lr}$ are the longitudinal tire stiffness of the front and rear wheels, respectively, and $s_{r}$,$s_{f}$ are the sliding rate of the front and rear tires, respectively. The experimental results show that the tire lateral force is linearly related to the side slip angle at little side slip angle condition, and the eligible linear tire model lateral force can be described as:

(4) $$\begin{array}{l} F_{cf}=C_{cf}\alpha_{f}\\ F_{cr}=C_{cr}\alpha_{r} \end{array}\}$$

where $C_{cf}$,$C_{cr}$ are the cornering stiffness of front and rear tires, respectively.

The front and rear wheel lateral deflection angle can be obtained from the following equation:

(5) $$\begin{array}{c} \alpha_{f}=\frac{\dot{y}+\dot{\varphi }a}{\dot{x}}-\delta_{f}\\ \alpha_{r}=\frac{\dot{y}-b\dot{\varphi }}{\dot{x}} \end{array}\}$$

where $\delta_{f}$ is the front steering angle. Bringing the above equations into derivation and simplifying them, the desired three-degree-of-freedom dynamics model is obtained as:

(6) $$\begin{array}{l} m\ddot{y}=-m\dot{x}\dot{\varphi }+2\left[C_{cf}\left(\delta_{f}-\frac{\dot{y}+a\dot{\varphi }}{\dot{x}}\right)+C_{cr}\frac{b\dot{\varphi }-\dot{y}}{\dot{x}}\right]\\ m\ddot{x}=m\dot{y}\dot{\varphi }+2\left[C_{lf}S_{f}+C_{lr}S_{r}+C_{cf}\left(\delta_{f}-\frac{\dot{y}+a\dot{\varphi }}{\dot{x}}\right)\right]\\ I_{z}\ddot{\varphi }=2\left[aC_{cf}\left(\delta_{f}-\frac{\dot{y}+a\dot{\varphi }}{\dot{x}}\right)-bC_{cr}\frac{b\dot{\varphi }-\dot{y}}{\dot{x}}\right]\\ \dot{Y}=\dot{x}\sin\varphi +\dot{y}\cos\varphi \\ \dot{X}=\dot{x}\cos\varphi -\dot{y}\sin\varphi \end{array}\}$$

The state space equations of the nonlinear dynamics model can be obtained by simple processing on the basis of the dynamics model:

(7) $$\begin{array}{l} \begin{array}{c} \ddot{y}=-\dot{x}\dot{\varphi }+\frac{2}{m}\left[C_{cf}\left(\delta_{f}-\frac{\dot{y}+a\dot{\varphi }}{\dot{x}}\right)+C_{cr}\frac{b\dot{\varphi }-\dot{y}}{\dot{x}}\right]\\ \ddot{x}=\dot{y}\dot{\varphi }+\frac{2}{m}\left[C_{lf}S_{f}+C_{lr}s_{r}+C_{cf}\left(\delta_{f}-\frac{\dot{y}+a\dot{\varphi }}{\dot{x}}\right)\right] \end{array}\\ \dot{\varphi }=\dot{\varphi }\\ \ddot{\varphi }=\frac{2}{I_{z}}\left[aC_{cf}\left(\delta_{f}-\frac{\dot{y}+a\dot{\varphi }}{\dot{x}}\right)-bC_{cr}\frac{b\dot{\varphi }-\dot{y}}{\dot{x}}\right]\\ \begin{array}{c} \dot{Y}=\dot{x}\sin\varphi +\dot{y}\cos\varphi \\ \dot{X}=\dot{x}\cos\varphi -\dot{y}\sin\varphi \end{array} \end{array}\}$$

## 3. Model Predictive Control Algorithm

Compared with nonlinear model predictive control, linear time varying model predictive control (LTV-MPC) has a higher computational speed and is more suitable for solving real-time path tracking problems. LTV-MPC minimizes the cost function by solving quadratic programming problem with multiple constraints and computing continuous control sequence in the prediction time domain. The solution process of the LTV-MPC algorithm can be generally divided into four steps. Firstly, by linearizing and discretizing the nonlinear continuous state model of the vehicle, the predicted state quantity and the predicted output quantity of the system are derived and sorted out; secondly, the reference path information is received to design the error-based cost function, and the cost function is transformed into the function to be optimized in the original primal problem of quadratic programming; then, the vehicle dynamics are analyzed and constraints are set by limiting the vehicle dynamics parameters; finally, a quadratic programming problem with inequality constraints is solved by using the quadratic programming solver.

### 3.1. State and Output Prediction

In the process of vehicle tracking, we need to predict the future state and output of the vehicle in a finite time horizon, and the accuracy of the prediction will directly affect the performance of the system when it works in practice.

The nonlinear dynamics model expressed by Equation (7) can be expressed as the following differential equation:

(8) $$\dot{\chi }(t)=f(\chi (t),u(t))$$

where $\chi (t)={\left[\dot{y},\dot{x},\varphi ,\dot{\varphi },Y,X\right]}^{T}$ is the state vector of the system and $u(t)=\delta_{f}$ is the input of the system. Linearizing it using Taylor’s method, we get:

(9) $$\dot{\chi }(t)=A_{t}\chi (t)+B_{t}u(t)$$

where $A_{t}$, $B_{t}$ is the Jacobian matrix and the two are calculated as:

(10) $$\begin{array}{c} A_{t}={\frac{\partial f(\chi (t),u(t))}{\partial \chi }|}_{\chi (t),u(t)}\\ B_{t}={\frac{\partial f(\chi (t),u(t))}{\partial \chi }|}_{\chi (t),u(t)} \end{array}\}$$

The discretization of $\dot{\chi }(t)$ using the forward Euler method is obtained as follow:

(11) $$\begin{array}{l} \chi (k+1)=A_{t}\chi (k)+B_{t}u(k)+d(k)\\ \eta (k+1)=C_{t}\chi (k) \end{array}\}$$

To introduce the control increment Δu, the original state vector is augmented. Let the new $\xi (k)={\left[\chi (k),u(k-1)\right]}^{T}$, to obtain the state space expression with Δu(k) as the input:

(12) $$\begin{array}{l} \xi (k+1)={\tilde{A}}_{k}\xi (k)+{\tilde{B}}_{k}\Delta u(k)+\tilde{d}(k)\\ \tilde{\eta }(k)={\tilde{C}}_{k}*\xi (k) \end{array}\}$$

where ${\tilde{A}}_{k}=\left[\begin{array}{cc} A_{k} & B_{k}\\ 0_{1\times 6} & 1 \end{array}\right]$, ${\tilde{B}}_{k}=\left[\begin{array}{c} B_{k}\\ 1 \end{array}\right]$, ${\tilde{C}}_{k}=\left[\begin{array}{c} C_{t}0_{2\times 1} \end{array}\right]$, $\tilde{d}(k)=\left[\begin{array}{c} d(k)\\ 0 \end{array}\right]$.

To facilitate the solution, the predicted output is expressed in the following matrix form:

(13) $$Y(k)=\Psi_{k}(k)\xi (k)+\Theta_{k}\Delta U(k)+\Gamma_{k}\Phi (k)$$

where

$$Y(k)=\left[\begin{array}{c} \tilde{\eta }(k+1)\\ \tilde{\eta }(k+2)\\ \vdots \\ \tilde{\eta }\left(k+N_{p}\right) \end{array}\right]{,\;\Psi }_{k}=\left[\begin{array}{c} {\tilde{C}}_{k}{\tilde{A}}_{k}\\ {\tilde{C}}_{k}{\tilde{A}}_{k}{}^{2}\\ \vdots \\ {\tilde{C}}_{k}{\tilde{A}}_{k}{}^{N_{p}} \end{array}\right]\;,\;\xi (k)=\left[\begin{array}{c} \chi (k)\\ u(k-1) \end{array}\right],$$

$$\Theta_{k}=\left[\begin{array}{cccc} {\tilde{C}}_{k}{\tilde{B}}_{k} & 0 & \ldots & 0\\ {\tilde{C}}_{k}{\tilde{B}}_{k} & {\tilde{C}}_{k}{\tilde{B}}_{k} & \ldots & 0\\ \vdots & \vdots & \ldots & \vdots \\ {\tilde{C}}_{k}{\tilde{A}}_{k}{}^{N_{p}-1}{\tilde{B}}_{k} & {\tilde{C}}_{k}{\tilde{A}}_{k}{}^{N_{p}-2}{\tilde{B}}_{k} & \ldots & {\tilde{C}}_{k}{\tilde{A}}_{k}{}^{N_{p}-N_{c}}{\tilde{B}}_{k} \end{array}\right]\;,\;\Delta U(k)=\left[\begin{array}{c} \Delta u(k)\\ \Delta u(k+1)\\ \vdots \\ \Delta u\left(k+N_{c}-1\right) \end{array}\right],$$

$$\Theta_{k}=\left[\begin{array}{cccc} {\tilde{C}}_{k}{\tilde{B}}_{k} & 0 & \ldots & 0\\ {\tilde{C}}_{k}{\tilde{B}}_{k} & {\tilde{C}}_{k}{\tilde{B}}_{k} & \ldots & 0\\ \vdots & \vdots & \ldots & \vdots \\ {\tilde{C}}_{k}{\tilde{A}}_{k}{}^{N_{p}-1}{\tilde{B}}_{k} & {\tilde{C}}_{k}{\tilde{A}}_{k}{}^{N_{p}-2}{\tilde{B}}_{k} & \ldots & {\tilde{C}}_{k}{\tilde{A}}_{k}{}^{N_{p}-N_{c}}{\tilde{B}}_{k} \end{array}\right]\;,\;\Delta U(k)=\left[\begin{array}{c} \Delta u(k)\\ \Delta u(k+1)\\ \vdots \\ \Delta u\left(k+N_{c}-1\right) \end{array}\right].$$

In the Equation (13), Y(k) is the output prediction matrix, $\Psi_{k}$ is the state coefficient matrix, $\Theta_{k}$ is the input coefficient matrix, $\Gamma_{k}$ is the system deviation coefficient matrix, Φ(k) is the system deviation matrix, $N_{P}$ is the prediction horizon of the algorithm, $N_{C}$ is the control horizon of the algorithm, and in general $N_{P}>N_{C}$ and $N_{P}\approx 3\sim 4N_{C}$.

### 3.2. Construction of Cost Function

The MPC algorithm tracking aims to make the difference between the predicted output and the reference value as small as possible, as this means that the vehicle can be tracked accurately following the intended path based on guaranteed lateral stability. Due to the complexity of the vehicle dynamics model and constraints, when the solver solves, the situation that the numerical solution cannot be obtained within one control cycle may arise, so a relaxation factor ℰ is introduced to make the algorithm run in the case of severe constraints can also obtain an executable solution [31].

Design the cost function as:

(14) $$\begin{array}{l} J(\xi (t),u(t-1),\Delta U(t))=\\ \overset{N_{P}}{\underset{i=1}{\sum }}{\left(y(t+i|t)-y_{ref}(t+i|t)\right)}^{T}\lambda_{y}\left(y(t+i|t)-y_{ref}(t+i|t)\right)+\\ \sum_{i=1}^{N_{P}}{\left(\varphi (t+i|t)-\varphi_{ref}(t+i|t)\right)}^{T}\lambda_{\varphi }\left(\varphi (t+i|t)-\varphi_{ref}(t+i|t)\right)+\\ \sum_{i=1}^{N_{c}-1}\Delta u{\left(t+i|t\right)}^{T}\lambda_{u}\Delta u(t+i|t)+\rho \varepsilon^{2} \end{array}$$

where J(ξ(t),u(t−1),ΔU(t)) is the sum of the costs, y(t+i|t), φ(t+i|t) and Δu(t+i|t) are the lateral position, heading angle and front steering angle increment at time t+i, respectively, $y_{ref}(t+i|t)$ and $\varphi_{ref}(t+i|t)$ are the reference lateral position and reference heading angle, respectively, $\lambda_{y}$, $\lambda_{\varphi }$ and $\lambda_{u}$ are the weight of the lateral error cost term, heading angle error cost term and front steering angle increment cost term in the corresponding objective function, respectively, and ρ is the relaxation factor weight. 

From Equation (14), the total cost is composed of three main components, the first part represents the cost of lateral error y, the larger the lateral error in the tracking process the higher the cost; the second part represents the cost of heading angle error φ, the larger the heading angle error in the tracking process the higher the cost; the third part represents the cost of front steering angle increment Δu, the larger the front steering angle increment in the tracking process the higher the cost.

Introducing E(k), E(k) is expressed in the following equation:

(15) $$E(k)=Y_{ref}(k)-\Psi_{k}\xi (k)-\Gamma_{k}\Phi (k)$$

Then, the cost function can be expressed as:

(16) $$J={\left[\Theta \Delta U-E\right]}^{T}\tilde{Q}[\Theta \Delta U-E]+\Delta U^{T}\tilde{R}\Delta U+\varepsilon^{T}\rho \varepsilon$$

where $\tilde{Q}$,$\tilde{R}$ are the weigh matrices. The cost function can be collated to obtain the original function to be optimized for which the quadratic programming can be solved:

(17) $$J=\frac{1}{2}{\left[\begin{array}{c} \Delta U(k)\\ \varepsilon \end{array}\right]}^{T}E\left[\begin{array}{c} \Delta U(k)\\ \varepsilon \end{array}\right]+F^{T}\left[\begin{array}{c} \Delta U(k)\\ \varepsilon \end{array}\right]$$

where

$E=\left[\begin{array}{cc} 2\left(\Theta^{T}\tilde{Q}\Theta +\tilde{R}\right) & 0\\ 0 & 2\rho \end{array}\right]$,$F^{T}=\left[\begin{array}{c} -2E^{T}(k)\tilde{Q}\Theta \;0 \end{array}\right]$.

### 3.3. Constraints

The establishment of constraints in model predictive control can reduce the deterioration of control system performance, reflecting the idea of optimal control, but if the conditions are too stringent, or even the number of linearly independent constraints exceeds the number of decision variables, then it will become difficult for the solver to perform the optimal solution. On the other hand, if the conditions are too loose, the probability that the algorithm will obtain a solution that violates the laws of physics to the extent that the performance deteriorates will be greatly increased and will not be able to achieve the effect of the constraint. For the consideration of actuator execution capability and system robustness, this paper mainly constrains the front steering angle and front steering angle increment.

The predicted front steering angle sequence $u_{k}\sim u_{k+Nc-1}$ can be expressed as follows:

(18) $$\begin{array}{c} u(k)=u(k-1)+\Delta u(k)\\ u(k+1)=u(k)+\Delta u(k+1)=u(k-1)+\Delta u(k)+\Delta u(k+1)\\ \vdots \\ u\left(k+N_{c}-1\right)=u\left(k+N_{c}-2\right)+\Delta u\left(k+N_{c}-1\right)=u(k-1)+\Delta u(k)+\Delta u(k+1)+\cdots \Delta u\left(k+N_{c}-1\right) \end{array}$$

The predicted sequence of front steering angle can be reduced to a form containing ΔU:

(19) $$U_{\;}=U_{t}+A_{I}\times \Delta U$$

where $U=\left[\begin{array}{c} u(k)\\ u(k+1)\\ \vdots \\ u\left(k+N_{c}-1\right) \end{array}\right]$, $U_{t}=\left[\begin{array}{c} u(k-1)\\ u(k-1)\\ \vdots \\ u(k-1) \end{array}\right]$, $A_{I}=\left[\begin{array}{cccc} I & 0 & 0 & 0\\ I & I & 0 & 0\\ \vdots & \vdots & \ddots & 0\\ I & I & \cdots & I \end{array}\right]$.

Then, the control input vector, i.e., the front steering angle, is constrained to be:

(20) $$\left[\begin{array}{c} u_{\min}\\ u_{\min}\\ \vdots \\ u_{\min} \end{array}\right]\leq \left[\begin{array}{c} u(k)\\ u(k+1)\\ \vdots \\ u\left(k+N_{c}-1\right) \end{array}\right]\leq \left[\begin{array}{c} u_{\max}\\ u_{\max}\\ \vdots \\ u_{\max} \end{array}\right]$$

Equation (20) can be expressed as:

(21) $$U_{\min}\leq U_{t}+A_{I}\times \Delta U\leq U_{\max}$$

where $u_{\max}$ and $u_{\min}$ are the maximum and minimum steering limits of the front wheels, respectively. By shifting $U_{t}$, we obtain the standard inequality form of the constrained front steering angle.

(22) $$\left[\begin{array}{cc} A_{I} & 0\\ -A_{I} & 0 \end{array}\right]\left[\begin{array}{c} \Delta U\\ \varepsilon \end{array}\right]\leq \left[\begin{array}{c} U_{\max}-U_{t}\\ -U_{\min}+U_{t} \end{array}\right]$$

Next, write the standard inequality form of constrained increment of the front steering angle.

(23) $$\left[\begin{array}{c} \Delta U_{\min}\\ 0 \end{array}\right]\leq \left[\begin{array}{c} \Delta U\\ \varepsilon \end{array}\right]\leq \left[\begin{array}{c} \Delta U_{\max}\\ M \end{array}\right]$$

where M is the upper limit of the relaxation factor. Combining the front steering angle constraint and the front steering angle increment constraint yields the constraint inequality required for the quadratic programming solution.

(24) AX≤b

### 3.4. Quadratic Programming Solution

Cost function Equation (17) and constraint Equation (24) constitute an original primal QP optimization problem. In general, the practical application of MPC algorithm is limited by the hardware arithmetic power, which is difficult to meet the requirements of processing online optimization problems, so it is important to choose an efficient and stable QP solver.

The original method for solving quadratic programming problems with constraints is the exhaustive method, i.e., a subset is selected from the entire set of constraints, defined as the current working set, and the suboptimization problem corresponding to that working set is solved. Although this violent search method can ensure that the solution of the optimal problem is obtained in a finite number of steps, the method will become very large in terms of operations when there are more constraints. Later, the active set method was proposed by Markowitz, which added the identification of optimal solutions according to the KKT condition and improved the search method of optimal solutions based on rules that make the cost function value decrease. This optimization dramatically reduces the workload of optimization. However, the active set method still has problems, such as cumbersome algebraic description and great computational effort, and the system is prone to no solution situations.

This control system uses the Hildreth real-time solver method for solving quadratic programming problems. The advantages are mainly the following: Firstly, the solver is based on the active set method by introducing the idea of dual, which is based on element-by-element search in finding the optimal decision variables without matrix inversion, so the computational space requirement is low. Secondly, when this solver has difficulty in searching for the optimal solution, it will provide a near-optimal solution, and will not consume a lot of time and space or even interrupt the program for exact convergence to a certain solution, which has good stability. Finally, because of the simplicity of the algebraic description of the Hildreth real-time solution method, it is more friendly for the development of MATLAB-based embedded automatic code generation [32,33,34].

The cost function obtained in Section 3.2 is combined with the constraints in Section 3.3 to obtain the following quadratic programming problem to be solved:

(25) $$\begin{array}{c} \underset{x}{\min}\left(\frac{1}{2}x^{T}Ex+F^{T}x\right)\\ s.t.Mx\leq \gamma \end{array}\}$$

This is also the primal problem of the dual problem, whose dual problem is

(26) $$\underset{\lambda \geq 0}{\max}\left(-\frac{1}{2}\lambda^{T}H\lambda -\lambda^{T}K-\frac{1}{2}F^{T}E^{-1}F\right)$$

where H and K are expressed as follows:

(27) $$\begin{array}{l} H=ME^{-1}M^{T}\\ K=\gamma +ME^{-1}F \end{array}\}$$

The global optimal solution $x=-E^{-1}F$ is calculated as the initial value $\lambda_{i}^{0}$, and then the matrix H, K is obtained by Equation (27). During the iteration, the elements in $\lambda^{m}$ are iterated one component at a time, and the iteration values follow the following equation:

(28) $$\begin{array}{c} w_{i}^{m+1}=-\frac{1}{h_{ii}}\left[k_{i}+\sum_{j=1}^{i-1}h_{ij}\lambda_{j}^{m+1}+\sum_{j=i+1}^{n}h_{ij}\lambda_{j}^{m}\right]\\ w_{i}^{m+1}=\max(0,w_{i}^{m+1)}) \end{array}\}$$

where the scalar $h_{ij}$ is the ij-th element in the matrix H, and $k_{i}$ is i-th element in the vector K, and $\lambda_{j}^{m}$, $\lambda_{j}^{m+1}$ denote the j-th Elements of λ after m and m+1 iterations, respectively.

Each iteration will change the individual component $\lambda_{i}$ in the Lagrangian coefficients, when the cost function can be considered as a quadratic function about the individual component $\lambda_{i}$, adjust $\lambda_{i}$ to minimize the cost function, and then consider the next component $\lambda_{i+1}$. It is generally considered that a complete cycle in which the solver converges by updating λ is one iteration of the vector $\lambda_{m}$ to $\lambda_{m+1}$. Finally, the iteration will be stopped when the result converges within the tolerable error or when the number of iterations exceeds the threshold.

Since the one-dimensional iterative technique in the Hildreth real-time solution method has been shown to converge to the set a and the set a that converges contains zero or positive values of Lagrange multipliers, solving for the decision variables of the primal problem yields the solution.

The decision variables of the primal problem are obtained from the following equation:

(29) $$x=-E^{-1}\left(F+M^{T}\lambda^{*}\right)$$

The pseudo-code for Algorithm 1, the Hildreth real-time solution, is as follows.
**Algorithm 1.** Hildreth′ s Quadratic Programming Algorithm 
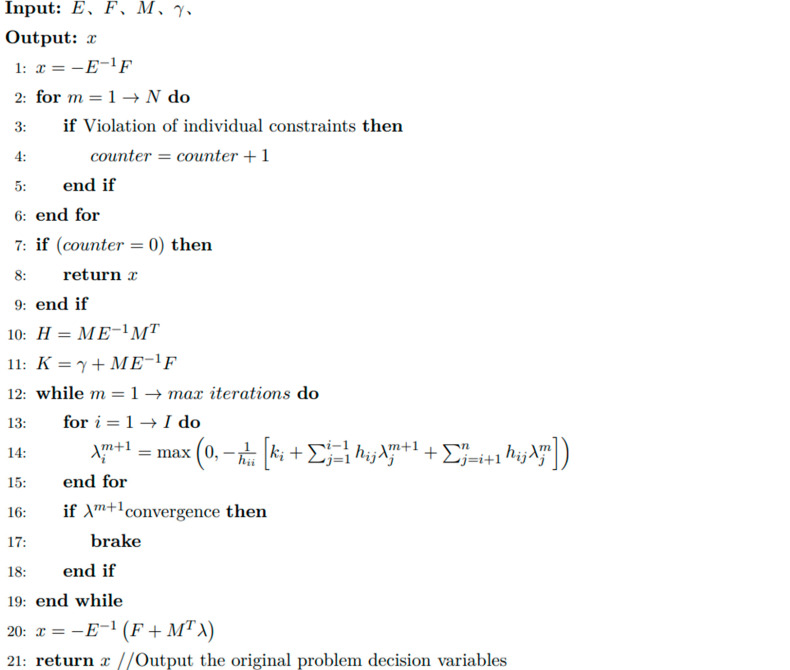


## 4. Weight Adaptive Optimization

### 4.1. Impact of Weights on Tracking Control

Some scholars usually set the weights in the cost function designed in Section 3.2 as constants, but in actual driving, due to different vehicle motion states and road environments, the emphasis on different parts of the cost in Equation (14) should be different. To investigate the influence of $\lambda_{y}$,$\lambda_{\varphi }$,$\lambda_{u}$ on the tracking control effect, a joint Prescan-Carsim-Simulink simulation platform is built, and a tracking straight road-large curvature curve scenario is designed, setting the road adhesion coefficient as 0.85 and the vehicle speed as 50 km/h, and there is a certain initial offset distance from the road centerline. Since the three weights in the cost function have relative significance, the experiments set a single weight in the cost function as a variable and the other two weights as constants, respectively, to analyze the effect of the weights on the effect of path tracking.

**Figure 2:**
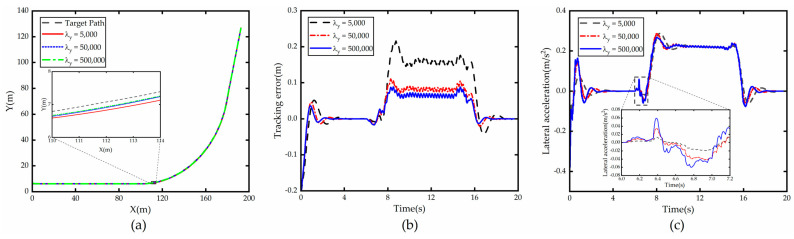
Comparison of path tracking under different $\lambda_{y}$. (**a**) Path; (**b**) Tracking error; (**c**) Lateral acceleration.

From Figure 2, which shows the comparison of simulation results with $\lambda_{y}$ as the variable, it can be seen that when $\lambda_{y}$ increases, the lateral error e generated by the vehicle tracking the road centerline decreases accordingly, which is due to the fact that increasing $\lambda_{y}$ makes the system more sensitive to the lateral error e generated at each time step, and the vehicle will quickly correct the error when e is generated. However, the larger the $\lambda_{y}$, the less obvious the reduction in e. However, the increase in $\lambda_{y}$ leads to the increase in lateral acceleration $a_{y}$ and the deterioration of stability.

**Figure 3:**
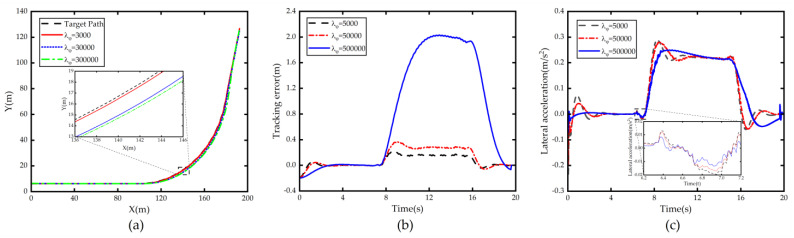
Comparison of path tracking under different $\lambda_{\varphi }$. (**a**) Path; (**b**) Tracking error; (**c**) Lateral acceleration.

From the simulation results of Figure 3, we can find that when $\lambda_{\varphi }$ increases, the lateral acceleration decreases and the stability is enhanced, but the lateral error e increases seriously, and when $\lambda_{\varphi }$ continues to increase, the lateral tracking error increases rapidly and the vehicle cuts out of this lane leading to tracking failure. This is due to the fact that when there is lateral error, excessive pursuit of the accuracy of tracking of φ will make the vehicle lose the ability to correct the error and tend to track parallel to the centerline of the lane with the existence of large error from the road centerline.

**Figure 4:**
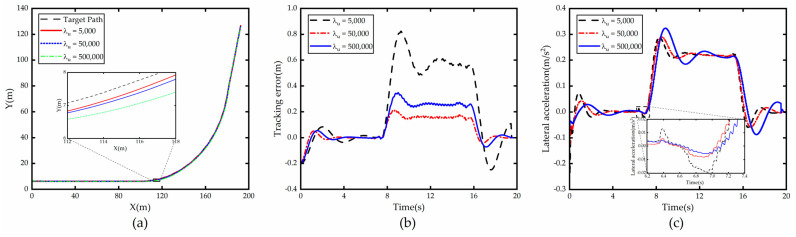
Comparison of path tracking under different $\lambda_{u}$. (**a**) Path; (**b**) Tracking error; (**c**) Lateral acceleration.

From the simulation results in Figure 4, it can be seen that when $\lambda_{u}$ increases, $a_{y}$ decreases slightly, but the lateral error e increases. This is due to the fact that increasing the penalty to the front steering angle increment causes the system’s sensitivity to lateral error and tracking heading angle to decrease, and the high-frequency jitter of the front steering angle gradually diminishes, which leads to the improvement of the stability.

In a comprehensive view, when tracking control of the path is performed by adjusting the weights, the two indicators, tracking accuracy and tracking stability, are mainly concerned, and the two are coupled, and the improvement of tracking accuracy will lead to the decrease in tracking stability, and vice versa. Further analysis, when more attention is paid to vehicle following, $\lambda_{y}$ should be increased to improve vehicle following, and when more consideration is given to driving stability, $\lambda_{\varphi }$ and $\lambda_{u}$ should be increased to improve stability, so the weights need to be adaptively adjusted according to the specific demand for tracking indexes when driving.

### 4.2. Weight Adaptive Analysis and Design

When tracking the straight road centerline, ensure the vehicle is smooth and comfortable, there is no need to enhance the tracking accuracy blindly, making the vehicle violently change the line. When there is a offset from the road centerline under a larger curvature curve from the vehicle, the driver’s intention to correct the offset is strong, with the aim of accurately tracking the road centerline and avoiding the vehicle cutting out of this lane, as the driver exists look-ahead, so the degree of preparedness for steering discomfort is reserved, at this time the driver will first ensure the tracking accuracy, and the comfort reduction becomes a tolerable item, so driving in large curvature curve should be adaptively adjusted weight to enhance the tracking of the vehicle to enhance the tracking accuracy, to avoid the driver panic because of the vehicle tracking ability is not enough to cut out the current lane.

According to the above analysis, in order to cope with the above situation, this paper adaptively adjusts the weight $\lambda_{y}$ based on the current vehicle speed and road curvature. When the road curvature is large, the weight $\lambda_{y}$ should be increased appropriately to enhance the following, and when the vehicle speed increases, the weight $\lambda_{y}$ should also be increased appropriately to alleviate the tracking lag caused by the vehicle inertia system.

**Figure 5:**
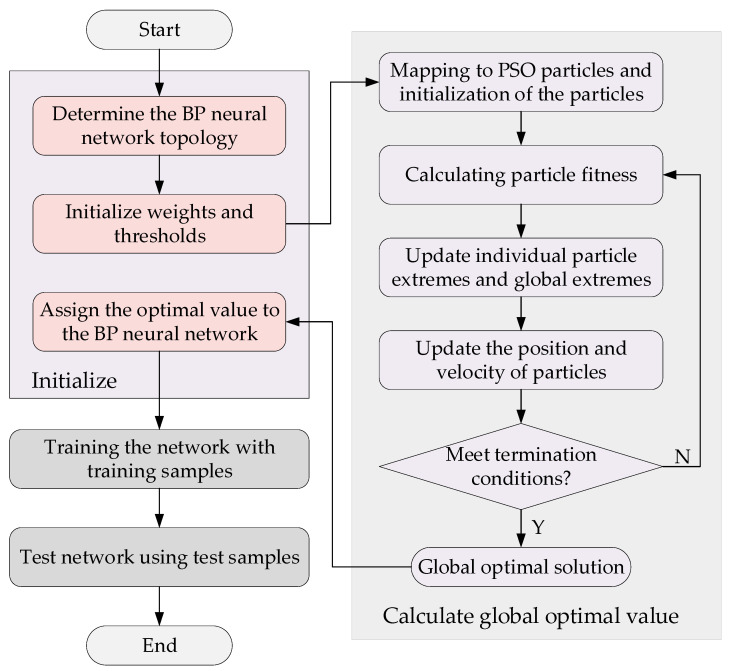
PSO-BO algorithm flow.

Since the analytic relationship between input and output is difficult to quantitatively describe, the BP neural network is selected for learning. BP neural network can learn and automatically extract the implicit mapping relationship between input and output data during training, it also can adaptively memorize the learning contents and store them in the network, i.e., BP neural network has a high degree of self-learning and self-adaptive ability, but due to the use of gradient descent method in error back propagation, it makes its solution easily fall into local optimum. Fortunately, the Particle Swarm Optimization (PSO) algorithm has a strong global search capability, and the global search capability of the PSO algorithm is used to optimize the connection weights and thresholds of the neural network, and the good global search capability of the PSO algorithm is combined with the good local search capability of the BP algorithm to improve the generalization capability and learning performance of the neural network, so as to improve the overall search efficiency of the neural network. The specific algorithmic flow of the neural network model is shown in Figure 5.

**Figure 6:**
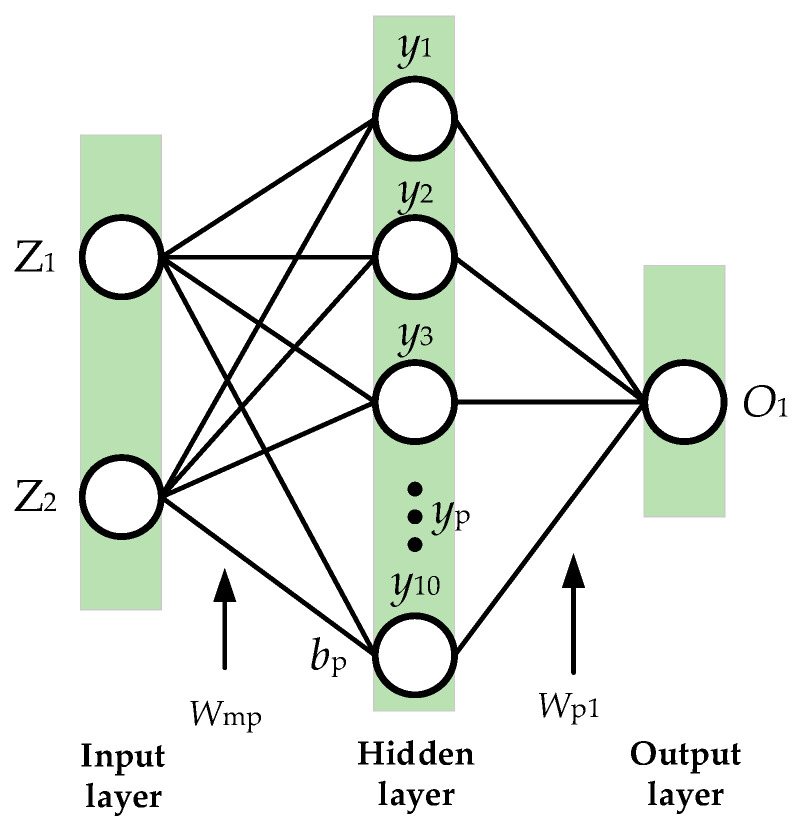
The structure of BP neural network.

A BP neural network based on PSO optimization algorithm is used for offline training of the weight model. In the training process, first of all, an automated simulation script is written based on MATLAB, and the optimal weights under different vehicle speeds and curvatures are obtained through a large number of automated simulation experiments to produce a dataset. The input dimension of BP neural network is 2, the number of hidden layer neurons is 10, and the output dimension is 1. The ratio of training set validation set test set is 7:1.5:1.5. The designed network structure is shown in Figure 6.

Then the parameters of the PSO optimization algorithm are designed, and the final parameters are determined as in Table 2.

**Figure 7:**
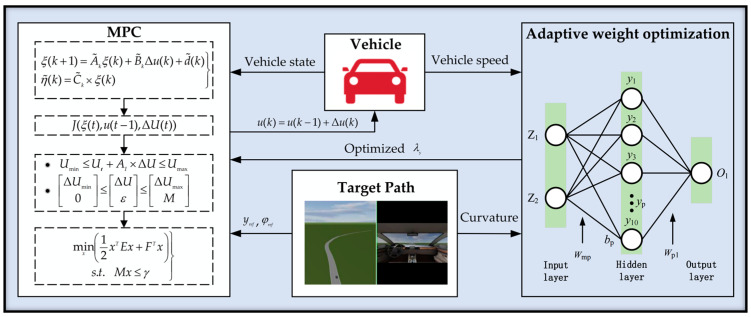
Overall architecture for path tracking.

The intelligent particles in the population then determine the initial hyperparameters of the BP neural network by continuously learning the historical data about themselves and the population. In this particular process, the PSO optimization algorithm first initializes the particle swarm and then determines the current adaptation value of each particle in accordance with the initial weights and biases that need to be set for the BP neural network. The data set is then input into the BP neural network for training, and the ideal PSO-BP neural network model is obtained by multiple training comparisons. After finishing the optimization, each particle’s velocity and position are updated according to the formula for the following iteration until the error is within the tolerance range. The adaptive method should be activated at a specific curvature and speed in order to adapt to complex road circumstances while improving robustness to changes in road characteristics fitted to the upstream lane line module. The trigger condition sets the absolute value of curvature in the range of 1/1000~1/30 and sets the speed range of 30~70 km/h, below or above which $\lambda_{y}$ is constant. The trained model receives the current road curvature and current vehicle speed and outputs the adaptive weight. Figure 7 is the overall block diagram of weight adaptive optimization path tracking algorithm.

## 5. Simulation

To verify the effectiveness of the proposed weight adaptive algorithm, a joint Prescan/Carsim/Simulink simulation platform is built to perform closed-loop simulation of autonomous vehicle, the simulation was performed with the following environment:

1. CPU: Intel Core i7-12700, 12Cores, 20 Threads, 4.90 GHz;
2. RAM: Kingston FURY 32.0 GB, DDR4 3200 MHz;
3. GPU: NVIDIA GeForce RTX 3060Ti, 8 GB DDR6;
4. Platform: Xingyun P3 platform, with the new Intel 600 series chipset;
5. Tool: Prescan8.5.0, Carsim2019.1; Matlab2020b.

The accurate model of vehicle dynamics is provided by Carsim, and the main parameters of the vehicle are shown in Table 3.

### 5.1. Simulation Results of Scenario 1

The experiment first designed a straight–semi-ring–straight road scenario, in which the length of the straight road section is 100 m, the radius of the semi-circular section is 50 m, set the road adhesion coefficient to 0.85, set the initial vehicle position and road centerline offset distance of 0.5 m, the vehicle speed is set to 40 km/h and 60 km/h, respectively, for simulation experiments. 

**Figure 8:**
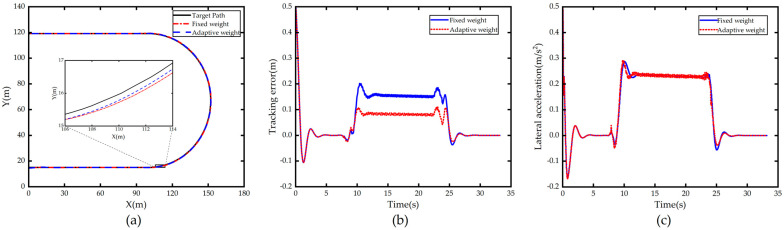
Comparison of 40km/h simulation results in Scenario 1. (**a**) Path; (**b**) Tracking error; (**c**) Lateral acceleration.

The simulation results for a constant speed of 40 km/h are shown in Figure 8. Figure 8a shows the comparison of the path tracking effect of the autonomous vehicle with the original controller and with the weight adaptive optimal control. It can be seen that both of them can track the path accurately and timely, but the tracking controller with weight adaptive optimal control performs better than the original controller in large curvature curves.

Figure 8b shows the comparison of tracking error, it shows that the improved controller has smaller tracking error on large curvature bends compared to the original controller, which is due to the fact that the trained neural network in the controller tends to output larger $\lambda_{y}$ when entering large curvature bends, and the system focuses more on following and tracking accuracy becomes better.

Figure 8c shows the lateral acceleration comparison, we can see that compared with the original controller, the lateral acceleration value of the optimized controller does not change much, and even decreases slightly when drive out of curve. This is mainly due to the fact that the vehicle tracking large curvature curve needs the front wheel angle deflection at high frequency to make the vehicle produce lateral displacement to reduce the tracking error when tracking the curve, and because the improved controller increases $\lambda_{y}$, the lateral error of the vehicle in the current moment is always maintained at a low value, which also leads to the vehicle using adaptive weight control without the need to quickly adjust the front wheel angle to control the vehicle in order to reduce the large lateral offset, so the value of the lateral acceleration caused by the front wheel angle is almost constant or even slightly reduced, and the stability is guaranteed.

**Figure 9:**
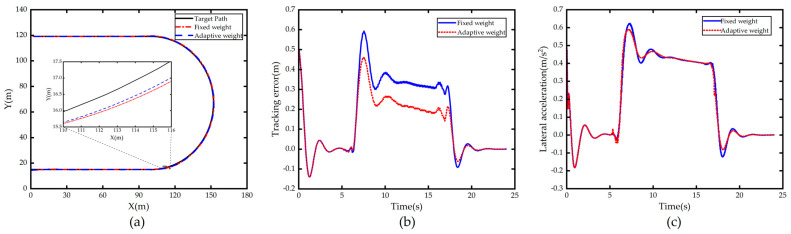
Comparison of 60 km/h simulation results in Scenario 1. (**a**) Path; (**b**) Tracking error; (**c**) Lateral acceleration.

The simulation results for a constant vehicle speed of 60 km/h are shown in Figure 9. From Figure 9a,b, it can be seen that the vehicle with weight adaptive optimal controller can track the curve more accurately and smoothly than the vehicle with fixed weight control, and the lateral tracking error is significantly reduced. However, compared to the speed of 40 km/h working conditions, the vehicle tracking error increased, especially when the lateral error from the straight road into the curve increased significantly, this is due to the straight road into the curve curvature oscillation, and the test road curvature reached 0.02, so the higher speed conditions into the large curvature curve when the outer drift is in line with the engineering reality.

From Figure 9c, it is known that the peak lateral acceleration of the vehicle after weight adaptive optimization is slightly reduced compared with that of the vehicle with fixed weight control, which is due to the fact that the variable weight controller increases $\lambda_{y}$ under large curvature conditions, making the MPC controller more sensitive to the error, and the tracking is better when it just enters the curved section, and the initial error of the vehicle tracking in the curved road is small, so there is no need to adjust the front steering angle significantly to compensate for the excessive initial error, then the lateral acceleration becomes smaller. In general, the weight adaptive optimization controller has a good tracking effect in both low and high-speed conditions.

### 5.2. Simulation Results of Scenario 2

**Figure 10:**
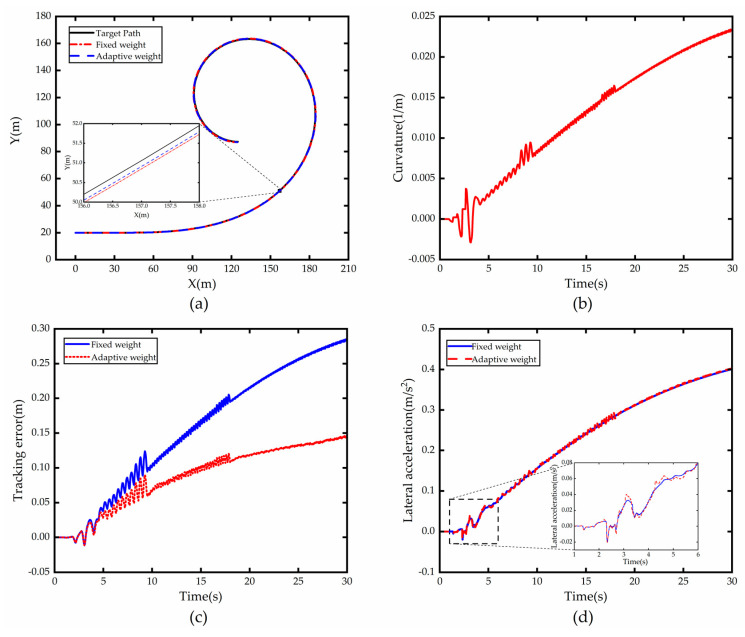
Comparison of 50 km/h simulation results in Scenario 2. (**a**) Path; (**b**) Curvature; (**c**) Tracking error; (**d**) Lateral acceleration.

The simulation of scenario 1 simulates the most common conditions in driving: keeping a straight line in the presence of initial offset and steering on a constant curvature curve with no initial offset, both types of roads with no change in curvature. It is also more common to steer on curves with variable curvature during daily driving, in order to further verify the performance of the controller in the variable curvature road, the clothoid curve road is selected for simulation verification. The clothoid curve is a curve in which the length and the radius of curvature are linearly related. The total length of the design scenario road is 421.7 m (including the initial section of 30 m straight road), the clothoid parameter is 115.08, no initial offset between vehicle and road, and the speed set to 50 km/h; clothoid road simulation results are shown in Figure 10.

Figure 10a shows the comparison of path tracking effect on clothoid road using fixed weight controller and after using weight adaptive optimal control; it can be seen that both of them can track the path accurately and timely, but the tracking controller after using weight adaptive optimal control performs better than the fixed weight controller on clothoid road section.

Figure 10b shows the road curvature information measured by the sensor when the autonomous vehicle is driving; it can be seen that the road is straight when the vehicle starts to drive from the starting point, and as the vehicle moves forward, there is a fitting error in the lane line output module from the time the sensor sensing range reaches the road junction to the time it travels over a straight section of road, this is due to the fact that polynomial methods cannot accurately fit the complete road shape for roads that are stitched together by straight roads and curved roads with variable curvature. The reason for the jitter in the curvature after driving into the clothoid curve road is that the performance of fitting higher order curves using third order polynomials is slightly inadequate, although the lane line module is self-limiting but generally satisfies the control requirements.

Figure 10c shows the comparison of the lateral error of vehicle tracking, and it can be seen that the tracking error of the vehicle increases gradually as the road curvature keeps increasing, but the error increases more and more slowly. The jitter vibration of the error is mainly due to the jitter of the actual measured road curvature of the vehicle. The comparison shows that the adaptive controller improves the tracking effect significantly compared with the original controller, which is due to the online adjustment of the weight adaptive controller according to the weights output from the PSO-BP neural network when tracking the road with variable curvature, which increases the $\lambda_{y}$, and as a result, it effectively suppresses the outward cutting phenomenon during vehicle tracking.

Figure 10d shows a comparison of the lateral acceleration of the vehicle, and it can be seen that both increase with the increase in road curvature. The lateral acceleration of the vehicle with the weight adaptive controller has a larger but roughly the same jitter compared to the lateral acceleration of the vehicle with the fixed weight controller, which is caused by the fact that the controller is more sensitive to errors after increasing $\lambda_{y}$.

The above simulation results can achieve effective tracking of paths. However, the weight adaptive optimization controller is able to track large curvature curves more accurately while ensuring vehicle stability, and performs well for straight roads, constant curvature curves and variable curvature curves.

## 6. Vehicle Testing and Analysis

**Figure 11:**
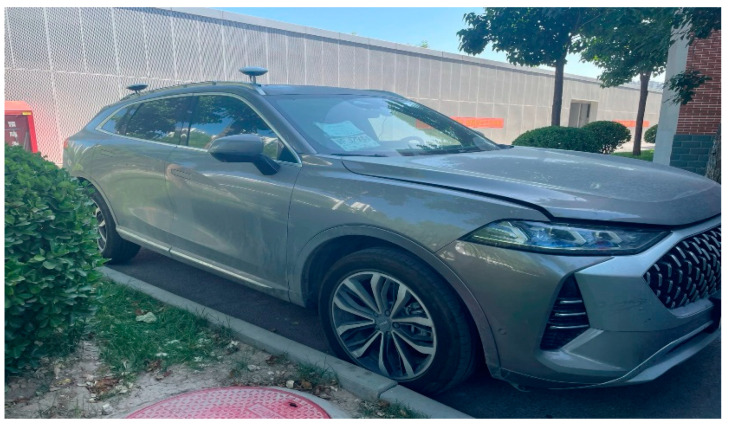
Autonomous vehicle test platform.

To further verify the effectiveness and real-time performance of the weight adaptive optimal control algorithm on the hardware platform, this paper conducts vehicle tests based on an autonomous vehicle platform. The autonomous vehicle test platform is shown in Figure 11.

**Figure 12:**
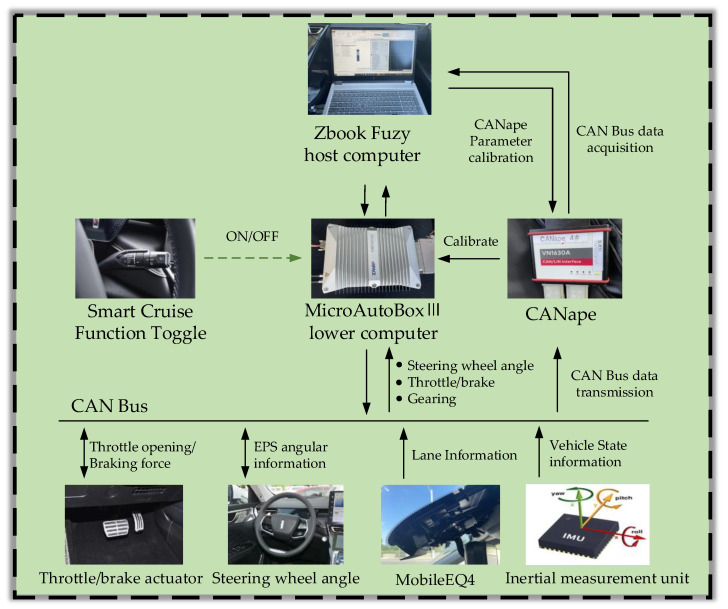
Path tracking control vehicle hardware system.

The test vehicle platform is a Great Wall Mocha SUV, and the vision front view sensor of the environment perception layer uses Intel Mobileye’s EyeQ4 solution (4th Generation), and the camera is installed on the front windshield to obtain lane information. The host computer on the control layer uses an HP Zbook fuzy mobile workstation with CANape18 for recording and storing operational data of the vehicle, and the lower computer is a MicroAutoBox III rapid prototyping real-time system from dSPACE, which transfers data between the host and lower computers via Ethernet. The actuation layer includes electronic throttle, electronic brake and electric power steering. The sensing layer includes a cornering sensor to detect the steering wheel corner and an inertial sensor to obtain the current vehicle motion. The base frequency within the vehicle software architecture is 100 Hz, the control module frequency is 50 Hz, the EPS adopts the corner control interface, data delay from control signal to EPS execution is no more than 200 ms, the workshop communication using CAN line transmission. The entire test platform hardware system is shown in Figure 12.

**Figure 13:**
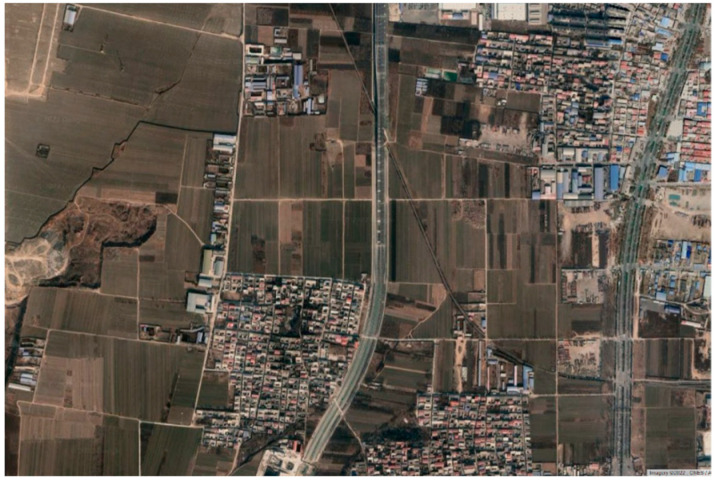
Satellite view of vehicle test road.

To test the algorithm, the algorithm was embedded in the ADAS framework control module for integration. The integrated ADAS software is compiled by the software dSPACE ConfigurationDesk developed by dSPACE in Paderborn, Germany, and the version number is 6.6. The compiled A2L file is then downloaded into the MicroAutoBox Ⅲ. During the vehicle test, when the driver confirms that there is no vehicle in front and the lane line is clear, the lever is toggled inward twice to activate the intelligent cruise function, at this time, the lateral movement is to achieve tracking control on the road centerline, and the longitudinal movement is for the driver to adjust the fixed speed through the button. Considering the safety of the open road and the requirements of traffic regulations, a section of a two-way expressway in Baoding, China, closed for construction in one direction was selected as the test road and the satellite map of the road is shown in Figure 13. The test road is about 1000 m long, including straight road section and variable curvature curve sections, the maximum curvature of the curve is about 0.00125, the test from south to north driving, the design maximum speed of 60 km/h, the driver can intervene at any time with the brake and steering wheel.

**Figure 14:**
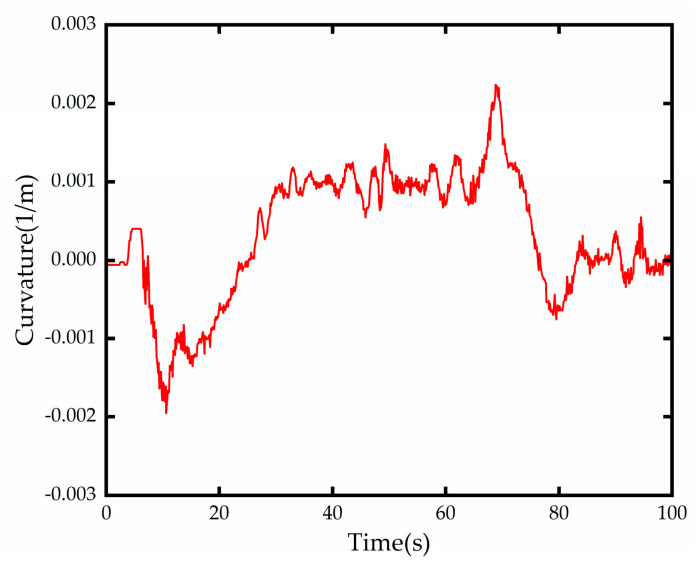
Road measured curvature change.

**Figure 15:**
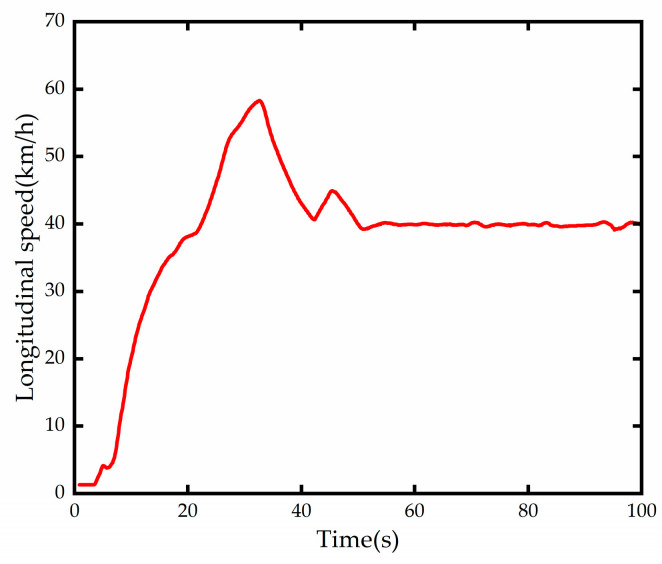
Longitudinal vehicle speed.

**Figure 16:**
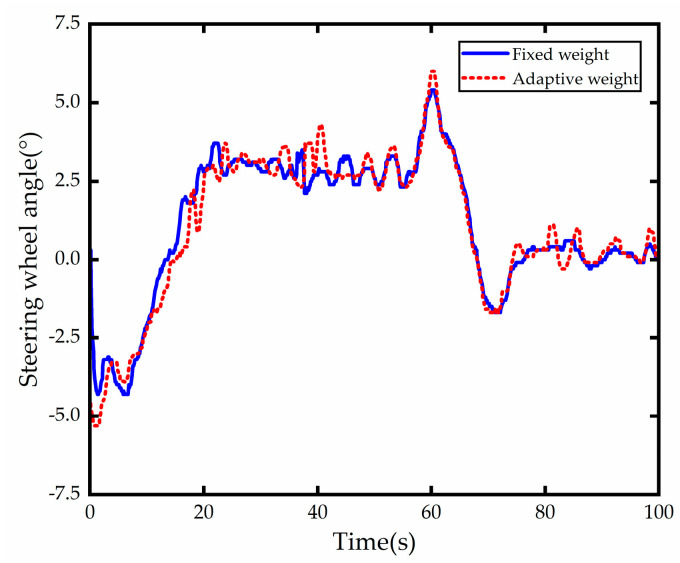
Steering wheel angle comparison.

**Figure 17:**
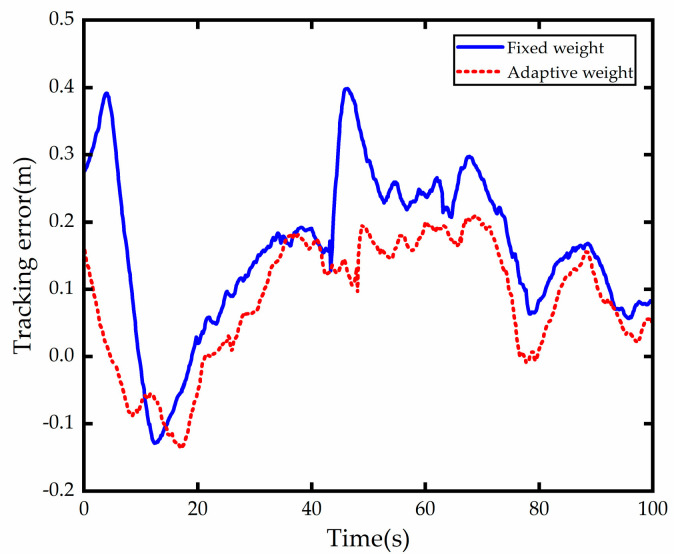
Tracking error comparison.

**Figure 18:**
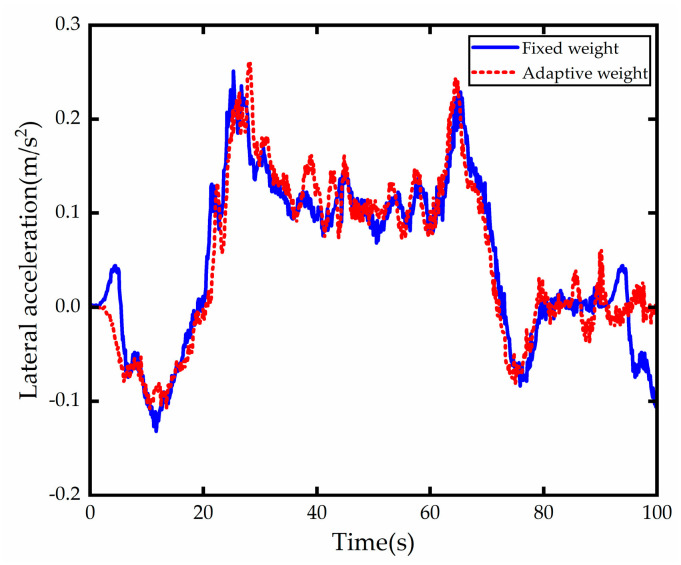
Lateral acceleration comparison.

The test needs to verify the effectiveness and robustness of the algorithm at different vehicle speeds and curvatures. The vehicle speed change mode is designed as follows: the vehicle speed value is set to 40 km/h at the beginning of 0s and the smart cruise function is activated, the speed is quickly adjusted to 60 km/h at 15 s and 40 km/h at 30 s, and then the current speed is kept constant until the end of the experiment. The data collected from the vehicle test and the comparison results of fixed weight optimization control and weight adaptive control are shown in Figure 14, Figure 15, Figure 16, Figure 17 and Figure 18.

The curvature of the test road obtained from the forward-looking lane line detection module is shown in Figure 14, and it can be seen that the variable curvature road has two continuous reverse curves with a minimum curvature radius of about 500 m. The longitudinal speed variation diagram is shown in Figure 15, and the test platform takes some time to reach the expected speed due to the existence of factors such as automatic gear lift and power system lag, but it does not contradict the test verification content.

The steering wheel angle values are shown in Figure 16, both control methods steering wheel angle are within a reasonable range and the steering wheel angle at the 9 s, 60 s, 71 s moments appear larger peak, produce steering wheel angle control amount of large jitter. The two curves in the figure demonstrate that at 40.5 s and 83 s, the vehicle using the adaptive weight control technique has a larger front wheel turning angle than the vehicle using the fixed weight control. This is because, compared to the tracking technique with fixed weight control, the tracking method with adaptive weight control imposes a larger weight, producing a higher increment of turning angle per unit time solved by its quadratic programming solver. Overall, both satisfy the actuator’s input requirements.

The road centerline trajectory tracking error results are shown in Figure 17; it can be seen that both methods can center the vehicle centerline, but the tracking accuracy with weight adaptive optimization control is significantly better than the tracking accuracy with fixed weight control. The first curve both performed better, with an error of about 0.12 m, and the vehicle with fixed weight control in the second curve deviates more from the centerline of the road when entering the curve, with a steady-state error of about 0.25 m in the lateral direction, while the vehicle with adaptive weight optimization shows better results in both the entry and steady-state turns, with a steady-state error of about 0.15 in the lateral direction, which is at most 60% better than the vehicle with fixed weight control.

According to the comparison of the lateral acceleration test results of the vehicle in the Figure 18, it can be seen that the lateral acceleration of the vehicle with adaptive weight control strategy at 40.5 s and 83 s increased than that of the vehicle with fixed weight control because of the increase in the change in front wheel turning angle. Although the lateral acceleration of the vehicle with adaptive weight optimization control was higher than that of the vehicle with fixed weight control, there was no apparent sudden change or jitter in the overall driving process. The difference between the test lateral acceleration of the vehicle with fixed weight control and that of the vehicle with fixed weight control was insignificant, this slight increase in acceleration was entirely acceptable for the driver.

## 7. Conclusions

To address the problem of insufficient tracking adaptability of autonomous vehicles under different vehicle speeds and road curvature, this paper proposes an adaptive path tracking method based on vehicle speed and road curvature based on model predictive control algorithm and trains the PSO-BP neural network model by data, which makes it possible to adjust the weight online for different working conditions. The necessity for a reliable and effective vehicle QP solver is met by the Hildreth real-time solver, which is conducive to real-car development. Vehicles are used to model and evaluate adaptive weight control, which improves tracking performance by up to 60% when compared to vehicles with fixed weight coefficient while maintaining vehicle stability standards. The proposed method has solid theoretical significance and engineering application value for the lateral control of intelligent vehicles. Later on, we will carry out more in-depth research on the adaptive motion control of autonomous vehicles with subjective driver evaluation and the robustness of vehicle lateral control algorithms under transient events, and the autonomous driving of industrial vehicles [35,36] is also one of our research directions in the future.

## Figures and Tables

**Table 1:** Definitions and symbols in dynamical model.

Description	Symbol	Unit
Vehicle mass	m	kg
Front wheel steering angle	$\delta_{f}$	rad
Side slip angle of front/rear tire	$\alpha_{f}/\alpha_{r}$	rad
Distance from c.g. to front/rear axle	a/b	m
Track width	c	m
Vehicle longitudinal speed (in xoy)	$\dot{x}$	m/s
Vehicle lateral speed (in xoy)	$\dot{y}$	m/s
Yaw rate	$\dot{\varphi }$	rad/s
Combined speed of front/rear tire	$V_{f}/V_{r}$	m/s
Left front/left rear/right front/right rear tire x-directional force	$F_{x,\mathrm{Lf}}/F_{x,\mathrm{Lr}}/F_{x,\mathrm{Rf}}/F_{x,\mathrm{Rr}}$	N
Left front/left rear/right front/right rear tire y-directional force	$F_{y,\mathrm{Lf}}/F_{y,\mathrm{Lr}}/F_{y,\mathrm{Rf}}/F_{y,\mathrm{Rr}}$	N
Longitudinal force of left front/left rear/right front/right rear tire	$F_{l,\mathrm{Lf}}/F_{l,\mathrm{Lr}}/F_{l,\mathrm{Rf}}/F_{l,\mathrm{Rr}}$	N
Lateral force of left front/left rear/right front/right rear tire	$F_{c,\mathrm{Lf}}/F_{c,\mathrm{Lr}}/F_{c,\mathrm{Rf}}/F_{c,\mathrm{Rr}}$	N

**Table 2:** PSO optimization algorithm parameters.

Parameter	Value
Learning factor	4.494
Number of population update	30
Population size	10
Maximum speed	1.0
Minimum speed	−1.0
Maximum boundary	1.0
Minimum Boundary	−1.0

**Table 3:** Parameters for simulations.

Vehicle Parameter	Value
Vehicle mass m/kg	1820
Distance from c.g. to front axle a/m	1.265
Distance from c.g. to rear b/m	1.682
$\mathrm{Vehicle}\;\mathrm{rotational}\;\mathrm{inertia}\;I/(\mathrm{kg}\cdot m^{2})$	4095
$\mathrm{Lateral}\;\mathrm{stiffness}\;\mathrm{of}\;\mathrm{front}\;\mathrm{tire}\;\alpha_{f}/(N/\mathrm{rad})$	87,508
$\mathrm{Lateral}\;\mathrm{stiffness}\;\mathrm{of}\;\mathrm{rear}\;\mathrm{tire}\;\alpha_{r}/(N/\mathrm{rad})$	65,317

## Data Availability

Not applicable.
